# Acetylcholine-mediated top-down attention improves the response to bottom-up inputs by deformation of the attractor landscape

**DOI:** 10.1371/journal.pone.0223592

**Published:** 2019-10-07

**Authors:** Takashi Kanamaru, Kazuyuki Aihara

**Affiliations:** 1 Department of Mechanical Science and Engineering, Kogakuin University, Tokyo, Japan; 2 International Research Center for Neurointelligence (WPI-IRCN), UTIAS, The University of Tokyo, Bunkyo-ku, Tokyo, Japan; 3 Institute of Industrial Science, The University of Tokyo, Meguro-ku, Tokyo, Japan; University of Michigan, UNITED STATES

## Abstract

To understand the effect of attention on neuronal dynamics, we propose a multi-module network, with each module consisting of fully interconnected groups of excitatory and inhibitory neurons. This network shows transitive dynamics among quasi-attractors as its typical dynamics. When the release of acetylcholine onto the network is simulated by attention, the transitive dynamics change into stable dynamics in which the system converges to an attractor. We found that this network can reproduce three experimentally observed properties of attention-dependent response modulation, namely an increase in the firing rate, a decrease in the Fano factor of the firing rate, and a decrease in the correlation coefficients between the firing rates of pairs of neurons. Moreover, we also showed theoretically that the release of acetylcholine increases the sensitivity to bottom-up inputs by changing the response function.

## Introduction

A large body of research demonstrates that neuromodulators play a major role in attention [1]. The role of acetylcholine (ACh), in particular, has attracted a great deal of attention.

Cholinergic cells in the nucleus basalis of Meynert (NBM) release ACh transiently to the cerebral cortex, and loss of these neurons is known to be associated with Lewy body dementia, the most salient symptom of which is recurrent complex visual hallucinations [2].

An auditory tone learning task showed that the strengths of synaptic currents in the auditory cortex of adult rats changed greatly when the tones were paired with activation of the NBM [3]. This result indicates that synaptic plasticity in the cortex is influenced by the ACh level.

When a cued appetitive response task was performed, in which rats were required to remember a light cue for several seconds to obtain reward, the concentration of ACh in the medial prefrontal cortex was observed to be increased while the rat maintained the cue in memory [4]. This result suggests that the concentration of ACh in the cortex increases when attention-demanding tasks are performed.

However, to our knowledge, no theories exist based on the dynamics of neural networks that account for the observed results of experiments of this type.

One of the most successful models of attention is the normalization model of attention [5–9]. In the normalization theory, responses of a neuron to sensory stimuli are divided by the summed activity of a pool of neurons; therefore, the response to a test stimulus often decreases when the contrast of another mask stimulus is increased. Attention works through this normalization mechanism and explains how the responses of a neuron to stimuli are modulated by attention.

The normalization model of attention is a powerful tool to understand the role of attention in visual recognition, but the mechanism of normalization is derived phenomenologically. We hope to understand the role of attention based on the nonlinear dynamics of networks composed of spiking neuronal models.

In this study, to understand the role of ACh released by attention, we examine the dynamics of a multi-module network, with each module consisting of fully interconnected groups of excitatory and inhibitory neurons. This model is based on a network model that we proposed in Kanamaru et al. [10].

When the network is in an attended state, ACh is released to it. The effects of ACh in the brain are controversial, and it is known to depend on several factors, such as the cortical depth and cell types [11]. Among the possibilities that have been described, we adopt the disinhibition of inhibitory synapses projecting onto excitatory neurons [12, 13].

We found that this model reproduces the results of the attention-dependent response modulation described by Mitchell et al. [14, 15]; while recording single-unit neural activity in V4 of macaques, they provided a visual stimulus in the receptive field of the observed neuron, and examined the differences between the response of the neuron to attended stimulation and that to unattended stimulation. The differences are summarized as follows.

1. The firing rate of a target neuron increased when the visual stimulus entered its receptive field. The firing rate increased further when the visual stimulus was attended than when it was unattended. The firing rates of inhibitory neurons were three times larger than those of the excitatory neurons [14].
2. The Fano factor of the firing rate decreased further when the visual stimulus was attended than when it was unattended. That is, the reproducibility of activity across trials increased for attended stimuli [15].
3. The correlation between the firing rates of pairs of neurons decreased further when the visual stimulus was attended than when it was unattended [15].

As for the effect of ACh, Linster and Hasselmo [16] examined the dynamics of a network composed of excitatory neurons and inhibitory neurons. They found that ACh enhances the response to sensory inputs by modulating excitatory synapses and inhibitory synapses [17]. Similarly, Deco and Thiele [18] also examined the dynamics of a network composed of excitatory neurons and inhibitory neurons. They introduced four effects of ACh into the network, *i.e*., a reduction in firing rate adaptation, an increase in thalamocortical synaptic efficacy, a reduction in lateral interactions, and an increase in inhibitory drive. They found that ACh modulates the difference of responses between attended states and unattended states. Their results explain the ACh-induced modulation of the firing rates of neurons.

Moreover, Deco and Hugues [19] proposed a neural network composed of excitatory neurons and inhibitory neurons to reproduce the decrease of the Fano factor using attention.

In addition to the attention-dependent response modulation, our model also reproduces the “contrast gain change” of the response function to external inputs, *i.e*., the leftward shift of the response function [1, 5–9]. Attention increases sensitivity of V4 neurons of monkeys by modulating the stimulus-response function in several ways [5, 6]. A change in the slope of the response curve is known as response gain change. An increase in the responses by a leftward shift of the nearly same response function is known as contrast gain change. These results are well-described by the normalized model of attention [5, 6].

In our network, we found that ACh increases the sensitivity of the network to external inputs by the contrast gain change, thereby providing a new interpretation of the contrast gain change from a viewpoint of nonlinear dynamics of neural networks.

The above results are consistent with the literature of the theory of attractors in nonlinear dynamics. With baseline levels of ACh, our previous network model exhibits transitive dynamics among quasi-attractors [10]. A quasi-attractor is a Milnor attractor in that there are positive-measure orbits approaching and temporarily persisting in state space [20]. However, a quasi-attractor may simultaneously possess repelling orbits. When the concentration of ACh in the network increases, each quasi-attractor is stabilized and the network converges to one of the stable conventional attractors. We called the arrangement of such attractors an attractor landscape, and we referred to the ACh-controlled deformation of the attractor landscape associated with attention as the “quasi-attractor hypothesis”. This network model was also intended to model experimental results for spontaneous activity in V2 of anesthetized cats with both eyes closed, reported by Kenet et al. [21]. Similar to the experiments of Kenet et al. [21], our previous model [10] did not have external inputs.

In the study of Thiele and Bellgrove [1], an attentional state, in which attention is focused on a target, is associated with a stable attractor and a less-focused state is associated with a transition among unstable attractors. If ACh can be assumed to control the attentional state, their view coincides with our quasi-attractor hypothesis [10].

In this study, we show that attention-dependent response modulation and change in response function can be reproduced by adding external inputs to our previous model. We also show that our results are consistent with both the quasi-attractor hypothesis [10] and the attention-dependent control of attractor states [1].

## Results

### Network structure

**Fig 1:**
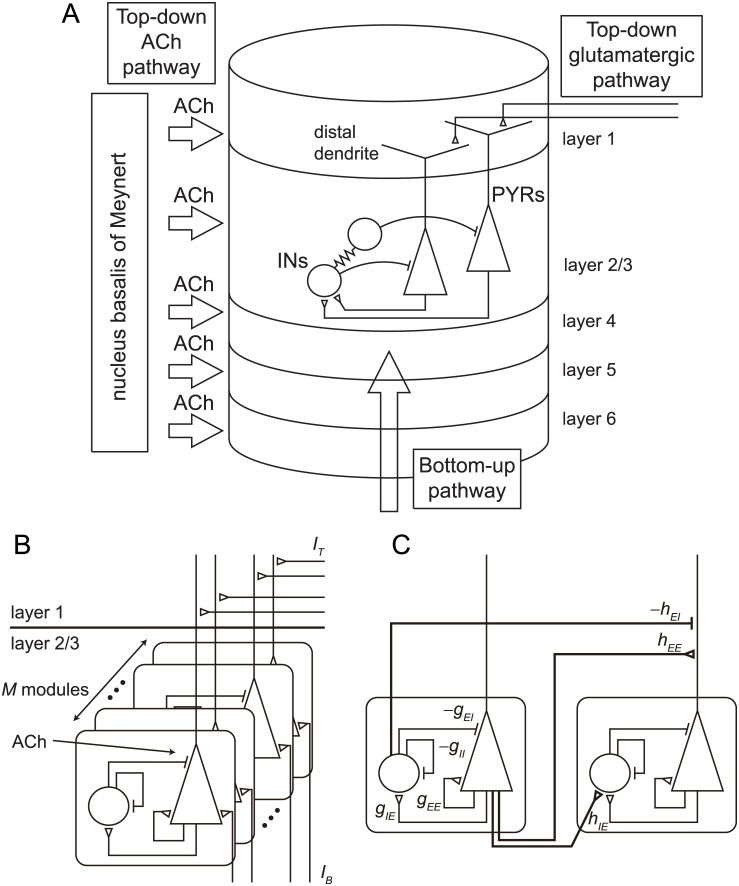
Network structure. (A) Layer structure of the cortex. There are three types of inputs in this model, *i.e*., cholinergic projections from the NBM, glutamatergic spike volleys from higher cortical areas and the thalamic matrix circuit, and a bottom-up external input injected via layer 4 from lower cortical areas. (B) *M*-module structure of a model. (C) There are four types of intra-module connections and three types of inter-module connections.

In this study, we examine a network composed of excitatory pyramidal neurons and inhibitory interneurons located in layer 2/3 of the cerebral cortex, as shown in Fig 1A.

There are three types of inputs in this model. First, cholinergic projections arise from the NBM [3, 22]. Second, glutamatergic spike volleys project to layer 1 from higher cortical areas and the thalamic matrix circuit [23, 24]. We regard these two inputs as top-down attentional inputs.

The third input is a bottom-up external input injected via layer 4 from lower cortical areas. This input corresponds to the input to the receptive field of the target neuron in the task that evokes attention-dependent response modulation [14, 15] or change in response function [1, 5–9].

In the following paragraphs, we examine the responses of the network to both top-down and bottom-up inputs.

We consider a multi-module network as a model of the network in layer 2/3 of the cortex [10]. A module is composed of *N*_*E*_ excitatory neurons and *N*_*I*_ inhibitory neurons. We regard this module as a model of a small network such as a pyramidal cell module [24] or a minicolumn [25]. In this type of module, various dynamic features such as stationary states, aperiodic firing, periodic synchronization, and chaotic synchronization can be observed [26].

Using module models, we defined a network composed of *M* modules located in layer 2/3, as shown in Fig 1B. We consider four types of intra-module connections, *E* → *E*, *E* → *I*, *I* → *E*, and *I* → *I*, and three types of inter-module connections, *E* → *E*, *E* → *I*, and *I* → *E*, as shown in Fig 1C, where *E* and *I* show groups of excitatory and inhibitory neurons, respectively. The detailed definition of connections is shown in the Methods section.

When the system is in an attended state, ACh is released to the cortex from the NBM. As stated in the Introduction section, we adopted the disinhibition of inhibitory synapses projecting onto excitatory neurons as an essential effect of ACh [12, 13]. We replaced the intra-module connection strength *g*_*EI*_ and the inter-module connection strength *h*_*EI*_ as shown in Fig 1C with *R*_*EI*_*g*_*EI*_ and *R*_*EI*_*h*_*EI*_, respectively, and we changed the degree of attention by regulating the inhibition rate *R*_*EI*_ in the range 0 < *R*_*EI*_ ≤ 1. Small *R*_*EI*_ corresponds to a strongly attended state.

In this study, we set *M* = 16, and the following three patterns are embedded in the connection weights of the network using the modified Hebbian rule [10]. Although we set the number *M* of modules and the number of patterns as small to reduce the computational time, a larger number of modules and patterns could be used as well in principle.

$$\begin{array}{ccc} \eta_{i}^{1} & = & \left\{ \begin{array}{cc} 1, & \text{if}\;i\leq M/2,\\ 0, & \text{otherwise,} \end{array}\right. \end{array}$$ (1)

$$\begin{array}{ccc} \eta_{i}^{2} & = & \left\{ \begin{array}{cc} 1, & \text{if}\;M/4<i\leq 3M/4,\\ 0, & \text{otherwise,} \end{array}\right. \end{array}$$ (2)

$$\begin{array}{ccc} \eta_{i}^{3} & = & \left\{ \begin{array}{cc} 1, & \text{if}\;i\;\text{mod}\;2=1,\\ 0, & \text{otherwise.} \end{array}\right. \end{array}$$ (3)

### Response of the network to top-down ACh

**Fig 2:**
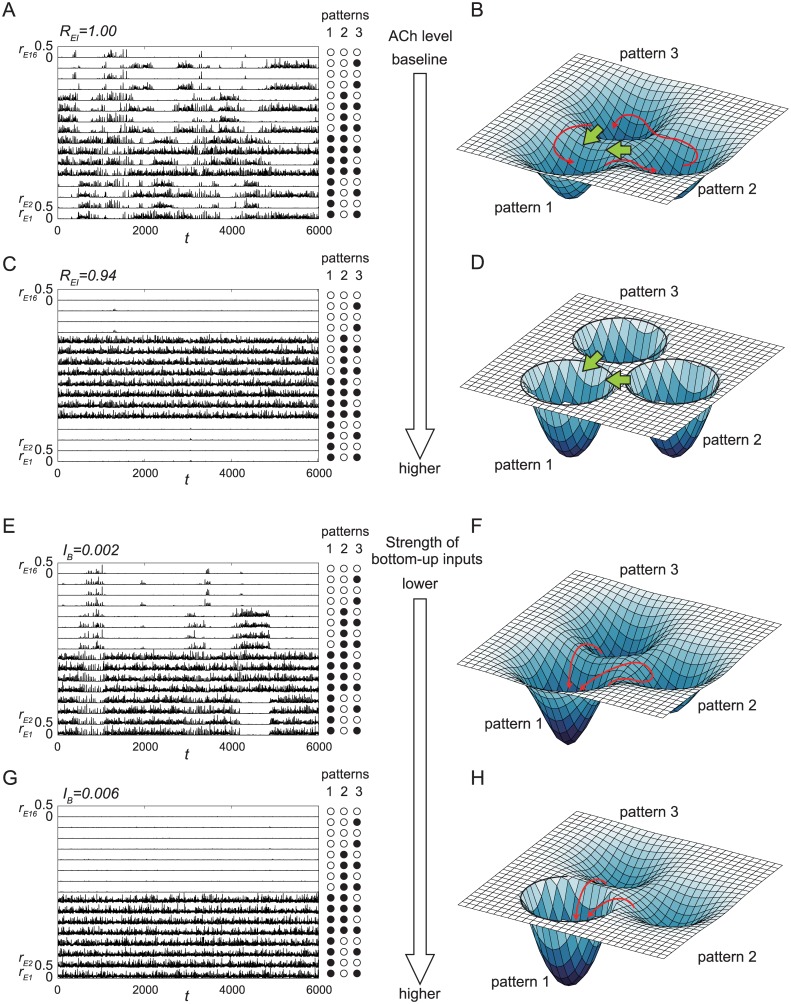
Relationship between network dynamics and deformation of the attractor landscape. (A-D) ACh-controlled deformation of the attractor landscape. When ACh is released, the inhibition rate *R*_*EI*_ decreases because of disinhibition of inhibitory synapses projecting onto excitatory neurons. (A, C) Dynamics of the network with 16 modules with *N*_*E*_ = 1000 and *N*_*I*_ = 250 for (A) *R*_*EI*_ = 1.00 and (C) *R*_*EI*_ = 0.94. (B, D) The corresponding attractor landscapes. The red arrows show the typical transitive dynamics. The green arrows show the influence caused by the glutamatergic spike volleys with strength *I*_*T*_. (E-H) Deformation of the attractor landscape caused by the bottom-up external inputs with strength *I*_*B*_. (E, G) Dynamics of the network with 16 modules with *N*_*E*_ = 1000 and *N*_*I*_ = 250 for (E) *I*_*B*_ = 0.002 and (G) *I*_*B*_ = 0.006. (F, H) Corresponding attractor landscapes. The red arrows show the typical transitive dynamics.

In this section, we analyze dynamics of a network of 16 modules with *N*_*E*_ = 1000 and *N*_*I*_ = 250. The total number of neurons is (1000 + 250) × 16 = 20000. The change in dynamics under the control of ACh as shown in Fig 2A, 2B, 2C and 2D is a typical example of the quasi-attractor hypothesis [10], and we explain this in detail in the following paragraphs.

The instantaneous firing rates *r*_*E*_ and *r*_*I*_ for excitatory and inhibitory ensembles in each module were calculated, and only *r*_*E*_ for 16 modules are shown in Fig 2A and 2C.

Fig 2A shows that each *r*_*E*_ has burst-like oscillations. Furthermore, the network reveals transitive dynamics among three patterns, which are shown in the right margin of Fig 2A. Both the oscillation of each module [26] and the global transitive dynamics [27] are chaotic. We regard such transitive dynamics as a resting or default-mode state [28] of the network.

A schematic diagram of this dynamics is shown in Fig 2B. The three stored patterns are shown as downward convex shallow potentials. Each potential is unstable, and the system can reach each pattern transiently and successively. Typical dynamics in this attractor landscape are shown as red arrows in Fig 2B. These unstable attractors were referred to as quasi-attractors in Kanamaru et al. [10].

Note that the degree of attention of the network is regulated by decreasing the inhibition rate *R*_*EI*_ from 1. The dynamics shown in Fig 2A were obtained with *R*_*EI*_ = 1; namely, the network is not in an attended state.

The attractors are stabilized as a result of the disinhibition. The dynamics for *R*_*EI*_ = 0.94 are shown in Fig 2C. The stability of each quasi-attractor increases and transitions among the patterns do not occur, as shown in Fig 2C. The corresponding attractor landscape is shown in Fig 2D, which also reveals that all of the attractors are stable. Note that the dynamics in each module are chaotic even when each attractor is stable as shown in Kanamaru et al. [10].

Note also that the attractor to which the system converges is not determined only by ACh. Fig 2C shows that the system converges to pattern owing to the initial state of the system. Therefore, a method is required that controls the system so that it can converge to the target attractor.

In Kanamaru et al. [10], to make the system converge to the target attractor, glutamatergic spike volleys projecting onto layer 1 from higher cortical areas and the thalamic matrix circuit were introduced to the model by adding constant inputs to the excitatory ensembles of modules corresponding to the target attractor with replacing the parameter *s*_*E*_ that determines the possibility of firing with *s*_*E*_ + *I*_*T*_. These are represented as the top-down pathway in Fig 1A and 1B. These top-down inputs cause the system to jump to other attractors along the green arrows shown in Fig 2B and 2D, and the system can converge to any attractor targeted when attention is strong enough.

As shown above, the quasi-attractor hypothesis is realized by the stabilization of attractors with ACh and the target attractor is selected by glutamatergic spike volleys [10].

### Response of the network to bottom-up inputs

In Kanamaru et al. [10], only the top-down inputs were composed of ACh, and the glutamatergic spike volleys were considered. In this section, we add bottom-up inputs to the network, and examine their effects.

As bottom-up inputs, we add constant inputs to the excitatory ensembles of modules *i* = 1, …, 8 by replacing the parameter *s*_*E*_ with *s*_*E*_ + *I*_*B*_. Note that in pattern 1, for example, the modules with 1 ≤ *i* ≤ 8 store “1”; therefore, the stability of pattern 1 would increase with positive *I*_*B*_.

The change in dynamics with the bottom-up inputs added is shown in Fig 2E, 2F, 2G and 2H.

While the release of ACh stabilized all the patterns, as shown in Fig 2D, the bottom-up inputs stabilize only pattern 1 as shown in Fig 2F and 2H, because the inputs are injected only to the modules with 1 ≤ *i* ≤ 8.

In Fig 1, we introduced three inputs to the network: the cholinergic inputs, the glutamatergic spike volleys, and the bottom-up inputs. In this study, the cholinergic inputs are globally applied to all the modules, and the glutamatergic spike volleys and the bottom-up inputs are locally applied only to some of the modules. Using these inputs, we reproduced the phenomenon of attention-dependent response modulation [14, 15] and the change of response function [1, 5–9].

### Response of the network to top-down and bottom-up inputs

In this section, we define inputs composed of top-down and bottom-up ones and we show typical responses of the network to the inputs.

**Fig 3:**
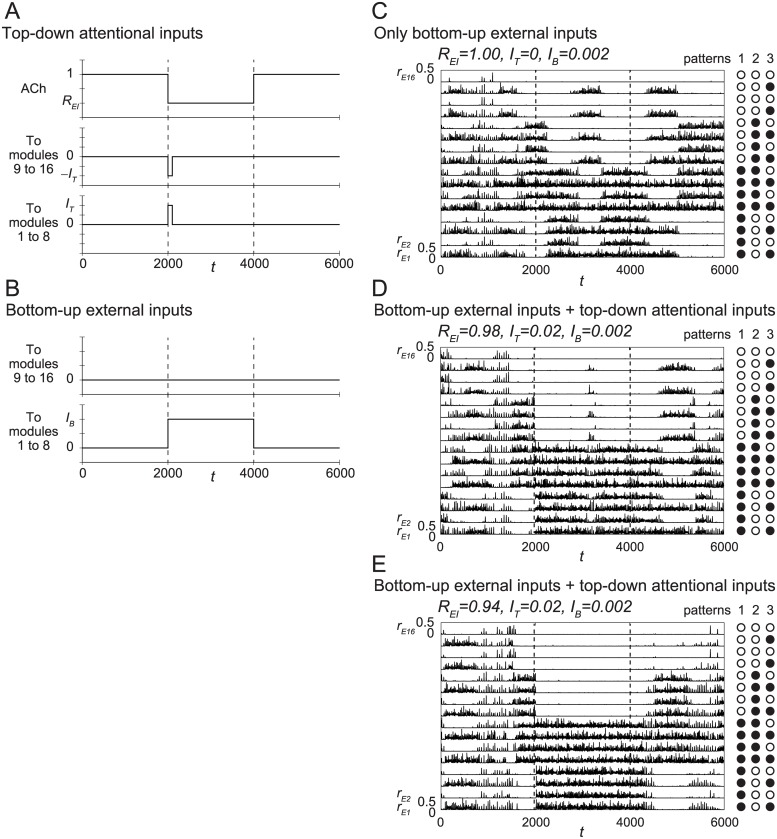
Responses of the network to inputs. (A, B) Inputs to the network. (A) Top-down attentional inputs composed of disinhibition of synapses caused by ACh and the glutamatergic spike volleys, which are controlled by *R*_*EI*_ and *I*_*T*_, respectively. (B) Bottom-up inputs are controlled by *I*_*B*_. (C-E) Attention-dependent changes in dynamics of the network. (C) Dynamics only with the bottom-up inputs for *R*_*EI*_ = 1, *I*_*T*_ = 0, and *I*_*B*_ = 0.002. (D, E) Dynamics with both the top-down attentional inputs and the bottom-up inputs for (D) *R*_*EI*_ = 0.98, *I*_*T*_ = 0.02, and *I*_*B*_ = 0.002 and (E) *R*_*EI*_ = 0.94, *I*_*T*_ = 0.02, and *I*_*B*_ = 0.002.

Numerical experiments were performed for 0 ≤ *t* ≤ 6000, and the three inputs shown in Fig 3A and 3B were injected into the network. Note that the inputs associated with attention were injected only during the period 2000 ≤ *t* ≤ 4000, and, during the remaining periods, the values of the parameters of the networks were identical to those used in Fig 2A.

In Fig 3A, two types of top-down inputs associated with attention are shown. First, the inputs associated with the release of ACh were applied to all 16 modules by decreasing *R*_*EI*_ from 1. With this input, the stabilities of all the patterns would increase as shown in Fig 2B and 2D.

Second, the top-down glutamatergic spike volleys were injected during the period 2000 ≤ *t* ≤ 2100 only. Positive pulses with strength *I*_*T*_ were applied to the excitatory neurons in the modules 1 ≤ *i* ≤ 8, and negative pulses with strength −*I*_*T*_ were applied to the excitatory neurons in the remaining modules. The negative pulses can be created by decreasing the total number of spikes arriving at the neurons during this short period. These inputs caused the system to transit to pattern 1 along the green arrows shown in Fig 2B and 2D.

Note that we can simulate a network without attention when we set *R*_*EI*_ = 1 and *I*_*T*_ = 0.

The bottom-up inputs are shown in Fig 3B. Positive inputs were injected only to the excitatory neurons in the modules 1 ≤ *i* ≤ 8. These inputs correspond to stimulation inside the receptive field in attention-related tasks. These inputs stabilize only pattern 1, as shown in Fig 2F and 2H.

The dynamics of the network with the inputs shown in Fig 3A and 3B is displayed in Fig 3C, 3D and 3E.

The dynamics of the network only with bottom-up inputs, *i.e*., *R*_*EI*_ = 1, *I*_*T*_ = 0, and *I*_*B*_ = 0.002 is shown in Fig 3C. Note that there are no top-down inputs for attention in the network because *R*_*EI*_ = 1 and *I*_*T*_ = 0, while the bottom-up inputs with *I*_*B*_ = 0.002 exist.

The time spent in pattern 1 was long for 2000 ≤ *t* < 4000, because of the positive *I*_*B*_ during this period. However, pattern 1 was still unstable. In other words, only the bottom-up inputs cannot stabilize pattern 1 completely. This dynamics corresponds to that shown in Fig 2F schematically.

The changes in the dynamics when the top-down inputs were added to this network are shown in Fig 3D and 3E.

The dynamics of the network with *R*_*EI*_ = 0.98, *I*_*T*_ = 0.020, and *I*_*B*_ = 0.002 is shown in Fig 3D. The system always transits to pattern 1 at *t* = 2000 because of the top-down glutamatergic spike volleys with *I*_*T*_ = 0.020. Pattern 1 is almost maintained during the period 2000 ≤ *t* < 4000 because of the inputs, but the pattern is still not fully stabilized. This dynamics also corresponds to that shown in Fig 2F.

The dynamics of the network with *R*_*EI*_ = 0.94, *I*_*T*_ = 0.020, and *I*_*B*_ = 0.002 is shown in Fig 3E. With these parameter values, pattern 1 is stabilized. This dynamics corresponds to that shown in Fig 2H.

In summary, the results in Fig 3C, 3D and 3E show that the top-down attentional inputs support the bottom-up inputs to stabilize a pattern.

### Statistics of firings of each neuron

Fig 3C, 3D and 3E show the temporal changes in instantaneous firing rates calculated from the firing times of all the excitatory neurons.

In this section, we obtain statistical data from the firing times of the neurons to compare the results with those of attention-dependent response modulation [14, 15].

**Fig 4:**
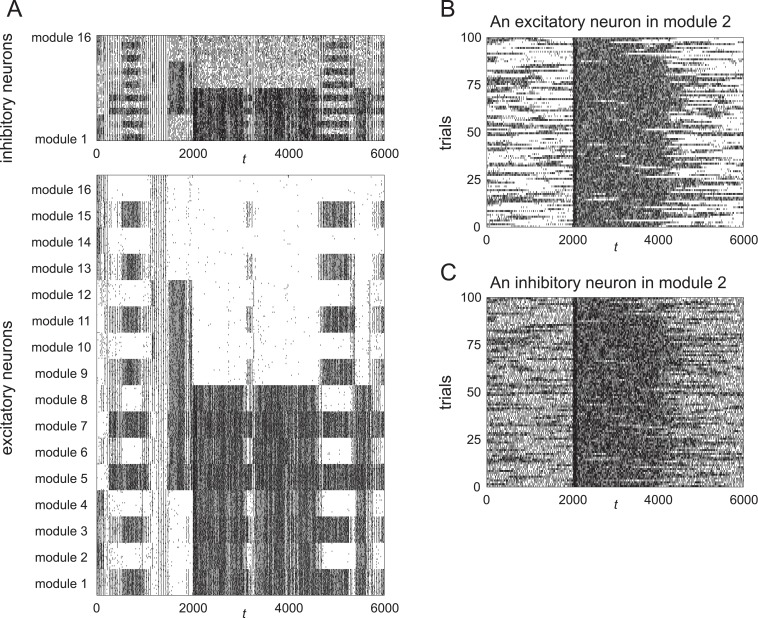
Spike activity of the neurons. (A) Raster plot of spike activity obtained from the dynamics for *R*_*EI*_ = 0.98 shown in Fig 3D. To reduce the volume of data, we show only the firings of 20 randomly chosen excitatory neurons and 5 randomly chosen inhibitory neurons in each module. (B) Spikes of a typical excitatory neuron in the second module for 100 simulated trials. (C) Spikes of a typical inhibitory neuron in the second module for 100 simulated trials.

First, in Fig 4A, we show a raster plot of the spike activity obtained from the data used in Fig 3D. To reduce the volume of data, only the data of 320 randomly chosen excitatory neurons and 80 randomly chosen inhibitory neurons are shown.

In the following, we examine only the activity of the neurons in the second module, because the second module stores “1” for pattern 1 and it stores “0” for patterns 2 and 3.

We performed 100 simulated trials, each of which yielded a raster plot similar to Fig 4A. We aligned the spikes of an excitatory neuron and an inhibitory neuron from the second module as shown in Fig 4B and 4C. It is evident that the firing times differ across trials, and the firing rates become large during the period 2000 ≤ *t* ≤ 4000 consistently over the trials.

Using data from 100 trials, we calculated the mean and the Fano factor of the firing count. First, we divided the time axis into bins with a period Δ*t* = 100. Using the number *μ*_*i*_(*j*) of firings in the *j*th bin for the *i*th trial, the mean firing count in the *j*th bin is defined as

$$\begin{array}{c} \bar{\mu }\left(j\right)=\frac{1}{N}\sum_{i=1}^{N}\mu_{i}\left(j\right), \end{array}$$ (4)

where *N* = 100. The variance of the firing count is defined as

$$\begin{array}{c} \sigma^{2}\left(j\right)=\frac{1}{N}\sum_{i=1}^{N}{\left(\mu_{i}\left(j\right)-\bar{\mu }\left(j\right)\right)}^{2}. \end{array}$$ (5)

The Fano factor in the *j*th bin is then defined as

$$\begin{array}{c} F\left(j\right)=\frac{\sigma^{2}\left(j\right)}{\bar{\mu }\left(j\right)}. \end{array}$$ (6)

### Attention-dependent response modulation caused by ACh

**Fig 5:**
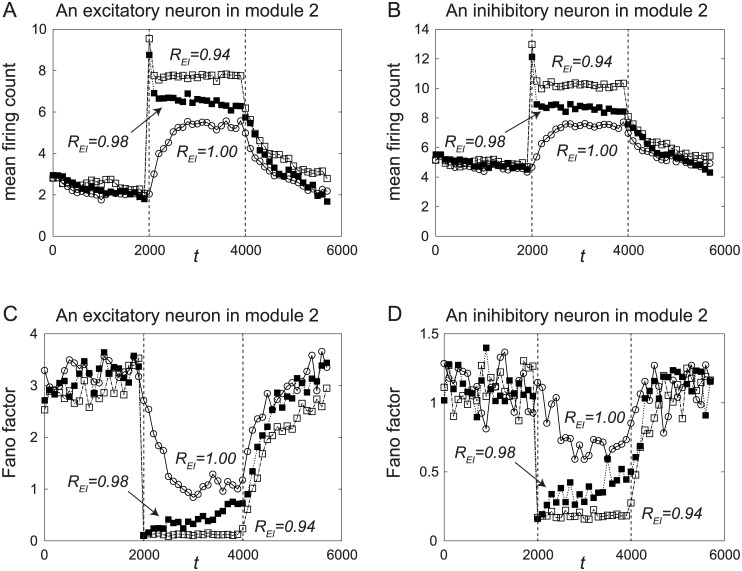
The mean firing count and its Fano factor. (A, B) Temporal changes in the mean $\bar{\mu }\left(j\right)$ of the firing count of an excitatory neuron and an inhibitory neuron in the second module. With the release of ACh, *i.e*., with the decrease in *R*_*EI*_, the firing rate increases. (C, D) Temporal changes in the Fano factor *F*(*j*) of the firing count of an excitatory neuron and an inhibitory neuron in the second module. With the release of ACh, the Fano factor decreases.

The mean $\bar{\mu }\left(j\right)$ and the Fano factor *F*(*j*) for an excitatory neuron and an inhibitory neuron in the second module are shown in Fig 5, where the horizontal axis shows *t* = *j*Δ*t*.

As in Fig 3C, 3D and 3E, the simulations were performed for *R*_*EI*_ = 1, 0.98, and 0.94. The bottom-up inputs with *I*_*B*_ = 0.002 are injected for all the simulations in this section. The top-down glutamatergic spike volleys with *I*_*T*_ = 0.020 are injected only when *R*_*EI*_ < 1. When *R*_*EI*_ = 1, we set *I*_*T*_ = 0. Therefore, the results for *R*_*EI*_ = 1 show the responses of the network to the unattended bottom-up inputs. When *R*_*EI*_ < 1, the bottom-up inputs are attended.

The temporal changes in mean firing count $\bar{\mu }\left(j\right)$ for an excitatory neuron and an inhibitory neuron are shown in Fig 5A and 5B, respectively. The mean firing count increased during the period 2000 ≤ *t* ≤ 4000, when ACh and the external signal were applied. This can also be seen in Fig 4B and 4C.

In addition, the mean firing count increases with the increase of ACh, *i.e*., and with the decrease of *R*_*EI*_. This happened because the probability of staying at pattern 1 becomes large when ACh is released, as shown in Fig 3C, 3D and 3E. Moreover, the firing count of the inhibitory neuron was larger than that of the excitatory neuron, as shown in Fig 5B.

Therefore, our results in Fig 5A and 5B reproduce the first property of the attention-dependent response modulation described in Introduction, *i.e*., the increase of the firing rate with the attended bottom-up inputs.

In our model, the firing count increases immediately at *t* = 2000 because the top-down glutamatergic spike volleys force the system to transit to pattern 1 along the green arrows in Fig 2B and 2D. However, in the experimental study, such an immediate increase of the firing count was not observed [14]. Therefore, in actual networks, there would be more gradual transitions of the system to other patterns.

In the theory of stochastic processes, it is known that the time scale of transitions among patterns is determined by the depth of the potential, in other words, the height of the potential barrier [29]. The release of ACh (the decrease of *R*_*EI*_) and the injection of bottom-up inputs deform the shape of the attractor landscape as shown in Fig 2. At *t* = 2000, the depths of all the potentials increase, and the deepest one is that of pattern 1. Therefore, the rate of transitions among patterns decreases. To cancel this effect, we used the top-down glutamatergic spike volleys with strong short pulses that do not change the shape of the attractor landscape, and they forcibly move the system to pattern 1. In order to realize the gradual transitions to pattern 1, the potentials of pattern 2 and pattern 3 should not be deepened. There would be two candidates for the gradual transitions to pattern 1: First, the top-down glutamatergic spike volleys with weak but longer pulses that would shallow the potentials of pattern 2 and pattern 3 in this period of pulses; and second, local ACh given only to the modules 1 ≤ *i* ≤ 8. Such local ACh would not deepen the potentials of pattern 2 and pattern 3. Details of applying such top-down inputs would be resolved in future studies.

The Fano factor *F*(*j*) decreased during the period 2000 ≤ *t* ≤ 4000, as shown in Fig 5C and 5D, and *F*(*j*) tended to decrease with increases in ACh, *i.e*., with decreases of *R*_*EI*_. This means that our results shown in Fig 5C and 5D reproduce the second property of the attention-dependent response modulation described in Introduction.

A small Fano factor means that the reproducibility of the activity across trials is high. This is related to the fact that the dynamics for *R*_*EI*_ = 1 is chaotically transitive. The transitive dynamics among patterns makes each module transit between burst-like activity and a nearly-silent state, as shown in Figs 3D and 4A. These transitions make the Fano factor large. In contrast, when ACh is released, the staying time at pattern 1 becomes longer, and the probability of occurrence of transitions among patterns becomes small; therefore, the Fano factor becomes small.

When pattern 1 is completely stabilized for *R*_*EI*_ = 0.94, the Fano factor takes a small constant value during the period 2000 ≤ *t* ≤ 4000, as shown in Fig 5C and 5D. However, in Mitchell et al. [14, 15], such major decreases of the Fano factor were not observed. Therefore, the pattern would not be completely stabilized in the experimental situations.

**Fig 6:**
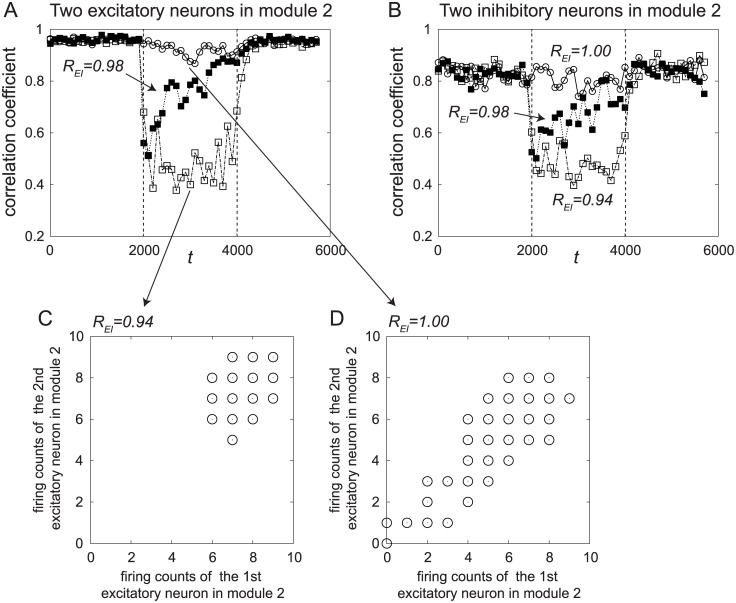
The correlation coefficients between the firing counts of pairs of neurons. (A, B) Temporal changes in the correlation coefficients of firing counts in the excitatory ensemble and the inhibitory ensemble in the second module. Two neurons were randomly chosen from each ensemble, and the correlation coefficient was calculated from data for 100 trials. (C, D) Distribution of the firing counts of two neurons, for (C) *R*_*EI*_ = 0.94 and (D) *R*_*EI*_ = 1.

Finally, we examined the correlation between firing counts of pairs of neurons. Two neurons from the excitatory ensemble or the inhibitory ensembles in the second module were randomly chosen, and the correlation coefficient of their binned firing counts was analyzed. The temporal changes in the correlation coefficients are shown in Fig 6A and 6B. The correlation coefficients decreased when ACh was released, that is, when *R*_*EI*_ decreased during the period 2000 ≤ *t* ≤ 4000.

This result might be counterintuitive, because when ACh is released, pattern 1 is stabilized and the spikes of neurons in the module might tend to chaotically synchronize with each other. However, we can understand the result by observing the distributions of firing counts of the two neurons.

The distribution of the firing counts of excitatory neurons at *t* = 3000 for *R*_*EI*_ = 0.94 is shown in Fig 6C. The dynamics of the network in this case shows chaotic synchronization in pattern 1, similar to the dynamics shown in Fig 3E around *t* = 3000. Therefore, the firing counts distribute in a small range, as shown in Fig 6C.

The distribution of the firing counts for unattended inputs, that is, for *R*_*EI*_ = 1, is shown at *t* = 3000 in Fig 6D. In this case, the second module shows transitive dynamics between burst-like firing and a nearly-silent state, as shown in Fig 3C. Therefore, the firing counts distribute in a wide range with a positive slope, as shown in Fig 6D, and a large correlation coefficient is obtained.

Therefore, the correlation coefficients decrease with an increase in the degree of attention. This result reproduces the third property of the attention-dependent response modulation described in Introduction.

### Comparison with Poisson spike train with inactive period

In the previous section, we found that the three properties of the attention-dependent response modulation are reproduced when transitive dynamics of the network disappears and a stored pattern is stabilized. In this section, we confirm that such properties are not obtained from stochastic firings based on the Poisson process.

A sequence of firing times {*t*_*i*_} (*i* = 0, 1, 2, …) of a Poisson spike train is obtained by calculating a difference equation

$$\begin{array}{c} t_{i+1}=t_{i}+T_{i}, \end{array}$$ (7)

where the inter-spike interval *T*_*i*_ obeys exponential distribution:

$$\begin{array}{c} f(T)=\lambda \exp(-\lambda T), \end{array}$$ (8)

with a firing rate λ.

We introduce an inactive period *T*_*r*_ to this sequence as

$$\begin{array}{c} t_{i+1}=\{\begin{array}{cc} t_{i}+T_{r}, & \text{if}\;T_{i}<T_{r},\\ t_{i}+T_{i}, & \text{otherwise.} \end{array} \end{array}$$ (9)

The inactive period *T*_*r*_ is the minimum length of the inter-spike interval. Typically, *T*_*r*_ is determined by a sum of the spike width and the refractory period of a neuron, *i.e*., *T*_*r*_ = 7 in our model. Moreover, when a neuron belongs to a module that shows synchronized oscillations, *T*_*r*_ tends to take larger values. For example, when a module shows a periodic synchronization, *T*_*r*_ is the period of such oscillation. Therefore, we also treat a case with *T*_*r*_ = 12.

Similar to Figs 5 and 6, we generate the sequences for 0 ≤ *t* ≤ 6000. In all the simulations, the firing rate is set to λ = λ_1_ = 0.02 during the periods 0 ≤ *t* ≤ 2000 and 4000 ≤ *t* ≤ 6000. During the period 2000 ≤ *t* ≤ 4000, we set λ = λ_2_, and λ_2_ is regulated to reproduce the firing rates in Fig 5A.

By generating 100 independent Poisson spike trains, we can calculate the firing counts and the Fano factors. By generating 100 pairs of independent Poisson trains, we can also calculate the correlation coefficients.

**Fig 7:**
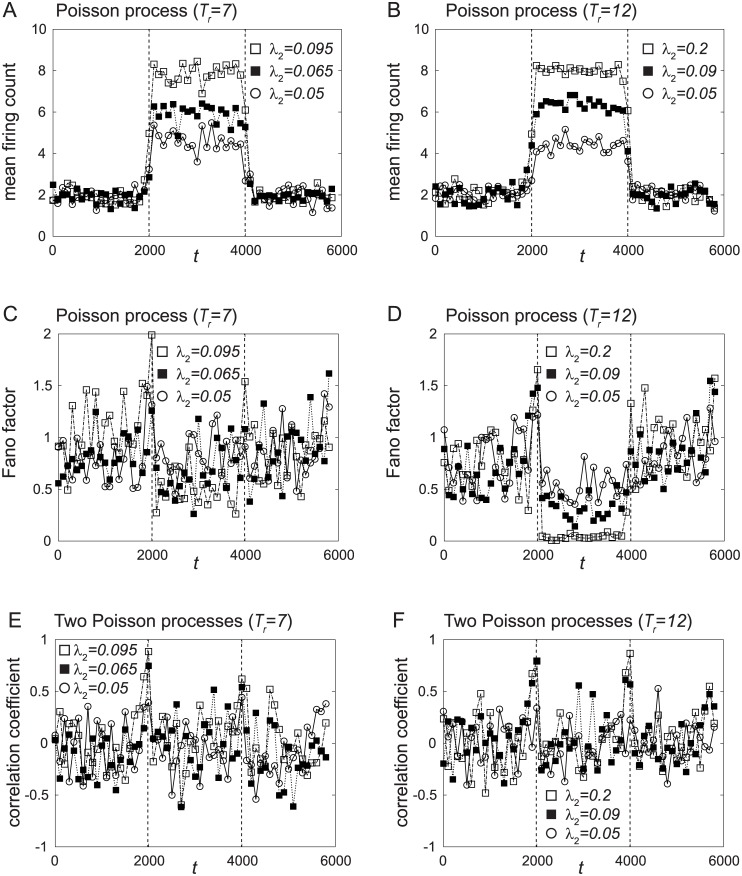
The mean firing count, the Fano factor, and the correlation coefficient of the Poisson spike trains with inactive period *T*_*r*_. (A, B) Temporal changes in the mean $\bar{\mu }\left(j\right)$ of the firing count for (A) *T*_*r*_ = 7 and (B) *T*_*r*_ = 12. The values of λ_2_ are chosen to reproduce the results in Fig 5A. (C, D) Temporal changes in the Fano factor *F*(*j*) of the firing count for (C) *T*_*r*_ = 7 and (D) *T*_*r*_ = 12. (E, F) Temporal changes in the correlation coefficient of the firing counts for (E) *T*_*r*_ = 7 and (F) *T*_*r*_ = 12.

In Fig 7A and 7B, the mean firing counts are shown for *T*_*r*_ = 7 and *T*_*r*_ = 12, respectively. The values of λ_2_ are chosen to reproduce the results in Fig 5A. Both results reproduce the results of Fig 5A well.

In Fig 7C and 7D, the Fano factors of the firing counts are shown for *T*_*r*_ = 7 and *T*_*r*_ = 12, respectively. When *T*_*r*_ is small (*T*_*r*_ = 7), the decrease of the Fano factor during the period 2000 ≤ *t* ≤ 4000 is small. When *T*_*r*_ = 12, the Fano factor decreases nearly to 0 for λ_2_ = 0.2. This result is similar to that of Fig 5C.

Therefore, we can conclude that the decrease of the Fano factor can be reproduced by introducing the inactive period *T*_*r*_ to the Poisson spike train. However, as shown in Fig 7E and 7F, the decrease of the correlation coefficient cannot be reproduced by the Poisson spike trains with *T*_*r*_.

Therefore, we propose that the three properties of the attention-dependent response modulation are caused by nonlinear dynamics in our network.

### Change of response function caused by ACh

In our network, the top-down ACh and the bottom-up inputs control the dynamical state of the network, *i.e*., they can deform the attractor landscape of the network as shown in Fig 2.

In the previous section, it was found that the top-down ACh causes the attention-dependent response modulation. In this section, we examine the role of the bottom-up inputs in the network. The inputs shown in Fig 3A and 3B are applied to the network.

**Fig 8:**
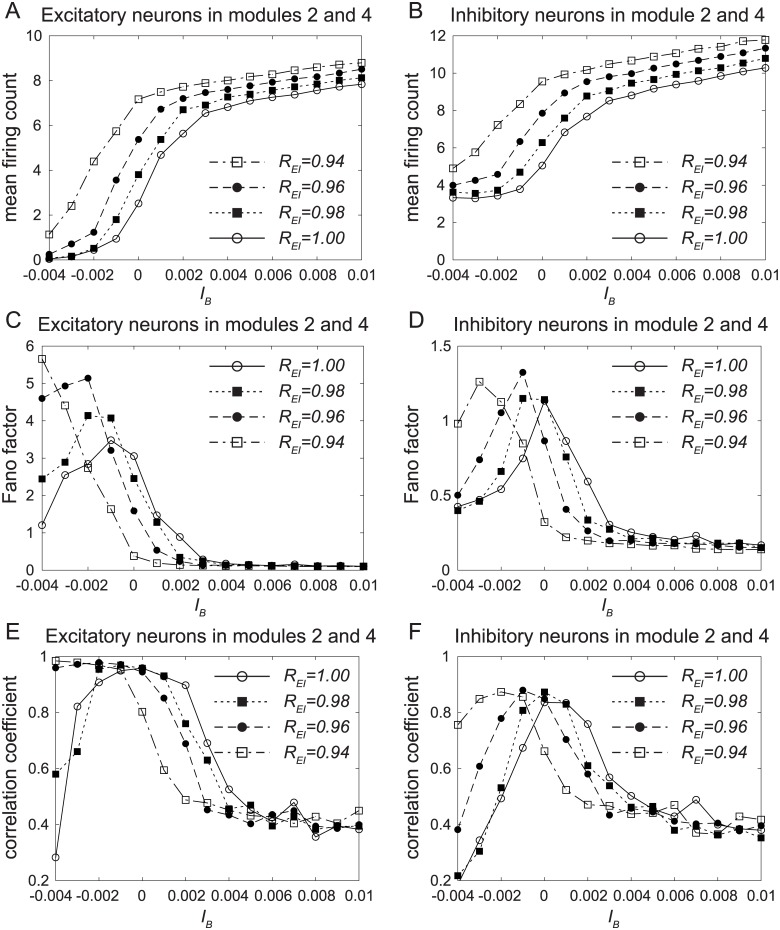
Responses of the network to the bottom-up input. The dependences of (A, B) the mean firing count, (C, D) the Fano factor, and (E, F) the correlation coefficient on the bottom-up input *I*_*B*_. The inputs shown in Fig 3A and 3B are applied to the network. The time-averaged statistics for 3000 ≤ *t* ≤ 4000 are calculated to discard transient dynamics for 2000 ≤ *t* ≤ 3000, and they are shown only for modules 2 and 4 because they store “1” for the pattern 1. The dependences of the firing count on *I*_*B*_ shown in (A) and (B) represent the response functions to the bottom-up input.

The dependences of the mean firing count, the Fano factor, and the correlation coefficient on the strength *I*_*B*_ of the bottom-up input are shown in Fig 8. Note that *I*_*B*_ can be interpreted as the contrast of visual stimuli.

The time-averaged statistics for 3000 ≤ *t* ≤ 4000 are calculated to discard transient dynamics for 2000 ≤ *t* ≤ 3000, and they are shown only for modules 2 and 4 because they store “1” for the pattern 1.

The dependences of the firing count on *I*_*B*_ shown in Fig 8A and 8B represent the response functions to the bottom-up input. Therefore, to observe the effect of ACh on the response function clearly, we performed simulations even for negative *I*_*B*_. Top-down attention increases the response to the bottom-up inputs by a leftward shift of the response function. In other words, top-down ACh in our model improves the sensitivity to the bottom-up inputs by the contrast gain change [1, 5–9]. Therefore, the top-down attention can be used as a support for the bottom-up inputs when the latter are not strong enough.

Note that our network shows some firing counts even when *I*_*B*_ = 0 as shown in Fig 8A and 8B. This is because we regard transitive dynamics shown in Fig 2A and 2B as a default-mode resting state of the network for *I*_*B*_ = 0.

Fig 8C, 8D, 8E and 8F show the dependences of the Fano factor and the correlation coefficient on *I*_*B*_. Similar to that of the top-down attention shown in Figs 5C, 5D, 6A and 6B, they decrease with the increase of *I*_*B*_ when *I*_*B*_ > 0. In addition, they decrease with the decrease of *I*_*B*_ in the range *I*_*B*_ < 0. In this range of *I*_*B*_, only the patterns 2 and 3 were stabilized.

## Discussion

In this study, we proposed a multi-module network that can reproduce the properties of the attention-dependent response modulation and change of response functions.

A module is composed of excitatory neurons and inhibitory neurons, and shows chaotic synchronization as its typical dynamics. After constructing a multi-module network as a model of the cortex, we embedded three patterns in the network and found transitive dynamics among quasi-attractors as the typical dynamics. To model the effects of ACh associated with attention, we adopted the disinhibition of inhibitory synapses projecting onto excitatory neurons. This disinhibition changes the dynamics of the network from the transitive regime to a stable regime in which the system converges to an attractor with a corresponding pattern. In addition to ACh, we used top-down glutamatergic spike volleys and bottom-up external inputs.

We found that this network could reproduce the three properties of the attention-dependent response modulation described in the Introduction [14, 15]. First, attention-dependent increases in firing rates are caused by an increase in the probability that the system stays in a stored pattern. Second, attention-dependent decreases in the Fano factors of the firing rates are caused by an increase in the reproducibility of the firing count with the stabilization of a stored pattern. Finally, attention-dependent decreases in correlation coefficients of firing rates are caused by shrinkage of the distribution of the firing counts into a narrow range. All these properties were caused by the change from transitive dynamics among patterns to stable dynamics in which the system converges to an attractor of a stored pattern, which was caused by the attention-dependent release of ACh.

Thus, these results indicate that the attention-dependent response modulation can be explained by the disinhibition of inhibitory synapses caused by the release of ACh. The top-down glutamatergic spike volleys and the bottom-up external inputs are used to specify the target pattern.

Moreover, we also found that our model reproduces the change in the response function of bottom-up inputs [1, 5–9]. The type of change in our model was the contrast gain change [1, 5, 6]. Reynolds and Heeger [8] found that attention with spatial dependence can yield various types of gain change. Conversely, ACh-mediated attention in our model was globally provided to the network. Thus, future studies should examine the spatial dependence of attention in our model.

In summary, top-down ACh in our model improves the sensitivity to the bottom-up inputs by increasing the firing rates and the contrast gain change of the response function. In other words, top-down attention can be used to support bottom-up inputs when they are not strong enough. This result is consistent with previous results of the normalized model of attention [5–9] and those of neural networks [16–18]. Moreover, our results suggest that such improvement in the response is accompanied by a decrease in the Fano factor and the correlation coefficient. The three properties of the attention-dependent response modulation and the contrast gain change of the response function are different aspects of a common phenomenon, *i.e*., change in the network dynamics from transitive dynamics among patterns to stable dynamics in which the system converges to an attractor of a stored pattern. Therefore, it is important to understand attentional phenomena from a standpoint of nonlinear dynamics of neural networks.

To model the effect of ACh in the network, we adopted the disinhibition of inhibitory synapses projecting onto excitatory neurons [12, 13]. However, several types of ACh effects are known which depend on factors, such as the cortical depth and cell types [11]. We regard that the increase of firing rates of excitatory neurons and associated stabilization of a pattern are essential in our results. Therefore, when these phenomena are kept unchanged, our results can also be reproduced by other effects of ACh. For example, Kanamaru et al. [10] examined dynamics of a network in which both excitatory and inhibitory synapses are modulated based on experimental results [30–35]. We found that the transitive dynamics and stabilization of stored patterns are also observed when modulations of excitatory and inhibitory synapses are balanced to some extent. Therefore, we expect that our results would be also observed even when different effects of ACh are adopted.

In our network, 50% of modules show firings when a pattern is stably retrieved as shown in Fig 2C and 2G, *i.e*., the activation rate of the modules is 0.5. It would be possible to observe similar results in a more “sparse” network with only a small number of modules show firings. However, to obtain such dynamics, the total number *M* of modules should be much larger than 16, and careful adjustment of parameters would be required. Therefore, we set the activation rate to 0.5 for simplicity.

In this study, our network has both top-down and bottom-up inputs, and they are injected to the network to obtain a common target pattern. When these inputs are injected to the network to obtain different patterns, a competition occurs. Typically, the pattern with stronger inputs is stabilized based on the winner-take-all mechanism. Such a competition also occurs when the bottom-up inputs are injected to the network to obtain two patterns. The top-down ACh can cause a bias to such a competition, *i.e*., ACh can explain the biased competition [36–38]. To analyze such dynamics, top-down ACh should be injected locally; however, it was injected globally to all the modules in this study. The role of such local top-down ACh will be analyzed in our future study.

The results obtained in this study are consistent with both the quasi-attractor hypothesis [10] and the attention-dependent control of attractor states [1]. Many attention-dependent properties are known in addition to the attention-dependent response modulation and gain change in response function [1]. Constructing a networks that can explain such properties and understanding them on the basis of dynamical systems theory are topics for future research.

## Methods

### Definition of a single module

As a model of the network in layers 2/3 of the cortex, we defined a module of a network to be composed of *N*_*E*_ pyramidal neurons and *N*_*I*_ interneurons, represented as phase neurons using the following equations:

$$\begin{array}{ccc} \tau_{E}\dot{\theta_{E}^{\left(i\right)}} & = & \left(1-\cos\theta_{E}^{\left(i\right)}\right)+\left(1+\cos\theta_{E}^{\left(i\right)}\right)\\ & & \times (s_{E}+\xi_{E}^{\left(i\right)}\left(t\right)+g_{EE}I_{E}\left(t\right)-g_{EI}I_{I}\left(t\right)), \end{array}$$ (10)

$$\begin{array}{ccc} \tau_{I}\dot{\theta_{I}^{\left(i\right)}} & = & \left(1-\cos\theta_{I}^{\left(i\right)}\right)+\left(1+\cos\theta_{I}^{\left(i\right)}\right)\\ & & \times (s_{I}+\xi_{I}^{\left(i\right)}\left(t\right)+g_{IE}I_{E}\left(t\right)-g_{II}I_{I}\left(t\right)+g_{gap}I_{gap}^{\left(i\right)}\left(t\right)), \end{array}$$ (11)

$$\begin{array}{ccc} I_{X}\left(t\right) & = & \frac{1}{2N_{X}}\sum_{j=1}^{N_{X}}\sum_{k}\frac{1}{\kappa_{X}}\exp(-\frac{t-t_{k}^{\left(j\right)}}{\kappa_{X}}), \end{array}$$ (12)

$$\begin{array}{ccc} I_{gap}^{\left(i\right)}\left(t\right) & = & \frac{1}{N_{I}}\sum_{j=1}^{N_{I}}\sin(\theta_{I}^{\left(j\right)}\left(t\right)-\theta_{I}^{\left(i\right)}\left(t\right)), \end{array}$$ (13)

$$\begin{array}{ccc} \left\langle \xi_{X}^{\left(i\right)}\left(t\right)\xi_{Y}^{\left(j\right)}\left(t^{\prime }\right)\right\rangle & = & D\delta_{XY}\delta_{ij}\delta \left(t-t^{\prime }\right), \end{array}$$ (14)

which have been used previously [10]. Each neuron model is referred to as a theta neuron [39]. The theta neuron has been used as a general model of a type-I spiking neuron [40, 41].

The activity of a single neuron in the model can be regulated by *s*_*X*_ (*X* = *E* or *I*). When *s*_*X*_ < 0, *θ* ∼ 0 is stable, this can be regarded as a resting state. When *s*_*X*_ > 0, the resting state becomes unstable, and *θ* starts to oscillate. The firing time is defined as the time at which $\theta_{X}^{\left(j\right)}$ exceeds *π*. In this study, we set *s*_*E*_, *s*_*I*_ < 0. Each neuron spontaneously fires with the help of Gaussian white noise $\xi_{X}^{\left(i\right)}\left(t\right)$ with the strength *D*.

The connections among neurons are global. Connections generating postsynaptic currents of an exponential form between all pairs of neurons as well as diffusive connections between inhibitory neurons are present. These two types of connections model chemical synapses and electrical synapses with gap junctions, respectively. $t_{k}^{\left(j\right)}$ is the *k*th firing time of the *j*th neuron. *g*_*EE*_, *g*_*IE*_, −*g*_*EI*_ and −*g*_*II*_ are connection weights within the excitatory and inhibitory ensembles, and *g*_*gap*_ is the strength of gap junctions. *τ*_*E*_ and *τ*_*I*_ are the membrane time constants, and *κ*_*E*_ are *κ*_*I*_ the time constants of synaptic currents.

The firing rates *r*_*E*_ and *r*_*I*_ of the excitatory ensemble and the inhibitory ensemble, respectively, are defined as

$$\begin{array}{ccc} r_{X}\left(t\right) & \equiv & \frac{1}{N_{X}w}\sum_{i=1}^{N_{X}}\sum_{l}\Theta \left(t-t_{l}^{\left(i\right)}\right), \end{array}$$ (15)

$$\begin{array}{ccc} \Theta (t) & = & \left\{ \begin{array}{cc} 1, & \text{for}\;0\leq t<w,\\ 0, & \text{otherwise,} \end{array}\right. \end{array}$$ (16)

where *X* = *E* or *I*, and *w* = 1.

### Connections among modules

To obtain a network of *M* modules, we defined the connection weights *T*_*Ei*_ and *T*_*Ii*_ of the input to the excitatory neurons and inhibitory neurons in the *i*th module [27] as

$$\begin{array}{ccc} T_{Ei} & = & \left(g_{EE}-g_{EE}^{sub}\right)I_{Ei}-\left(g_{EI}-g_{EI}^{sub}\right)I_{Ii}+\sum_{j=1}^{M}h_{ij}^{EE}I_{Ej}+\sum_{j=1}^{M}h_{ij}^{EI}I_{Ij}, \end{array}$$ (17)

$$\begin{array}{ccc} T_{Ii} & = & \left(g_{IE}-g_{IE}^{sub}\right)I_{Ei}-g_{II}I_{Ii}+\sum_{j=1}^{M}h_{ij}^{IE}I_{Ej}, \end{array}$$ (18)

where *I*_*Ei*_ and *I*_*Ii*_ are the sums of postsynaptic currents of the *i*th module defined by Eq 12. We replaced the inputs to the excitatory neurons and inhibitory neurons used in Eqs 10 and 11 with *T*_*Ei*_ and *T*_*Ii*_.

In the above definitions, the intra-module connection weights *g*_*EE*_, *g*_*EI*_, and *g*_*IE*_ are decreased by subtracting $g_{EE}^{sub}$, $g_{EI}^{sub}$, and $g_{IE}^{sub}$, respectively, to maintain the chaotic dynamics observed in a module.

The inter-module connection weights $h_{ij}^{EE}$, $h_{ij}^{EI}$, and $h_{ij}^{IE}$ are defined based on a modified Hebbian rule as follows:

$$\begin{array}{ccc} h_{ij}^{EE} & = & \left\{ \begin{array}{cc} h_{EE}K_{ij}, & \text{if}\;K_{ij}>0,\\ 0, & \text{otherwise}, \end{array}\right. \end{array}$$ (19)

$$\begin{array}{ccc} h_{ij}^{EI} & = & \left\{ \begin{array}{cc} 0, & \text{if}\;K_{ij}>0,\\ h_{EI}K_{ij}, & \text{otherwise}, \end{array}\right. \end{array}$$ (20)

$$\begin{array}{ccc} h_{ij}^{IE} & = & h_{IE}\left|K_{ij}\right|, \end{array}$$ (21)

$$\begin{array}{ccc} K_{ij} & = & \frac{1}{Ma(1-a)}\sum_{\mu =1}^{p}\left(\eta_{i}^{\mu }-b\right)\left(\eta_{j}^{\mu }-a\right), \end{array}$$ (22)

where $\eta_{i}^{\mu }\in \left\{0,1\right\}$ are stored patterns with the firing rate *a* = 0.5, *h*_*EE*_ = 3.0, *h*_*IE*_ = 1.55, and *h*_*EI*_ = 0.1. In the conventional associative memory model, *b* is set identical to *a* in Eq 22; however, we use *b* as a regulating parameter because our model differs from the conventional model, such as the inhibition realized by inhibitory ensembles, and we set *b* = 0.55.

When *K*_*ij*_ > 0, there are two types of inter-module connections, *i.e*., *E* → *E* and *E* → *I*, and such connections tend to induce inter-module synchronization. Conversely, when *K*_*ij*_ < 0, the connections *I* → *E* and *E* → *I* exist, and such connections tend to break the inter-module synchronization.

Three additional parameters of regulation, $g_{EE}^{sub}$, $g_{EI}^{sub}$, and $g_{IE}^{sub}$, are respectively defined as $g_{EE}^{sub}=\gamma h_{EE}$, $g_{EI}^{sub}=\gamma h_{EI}$, and $g_{IE}^{sub}=\gamma h_{IE}$ using a new parameter *γ* = 0.75 that is common to all modules. These are introduced to our model to maintain the chaotic dynamics observed in a one-module system with *g*_*EE*_, *g*_*IE*_, and *g*_*EI*_. Without them, the chaotic dynamics are broken, and periodic dynamics or asynchronous firing would be observed.

The values of the parameters used in a module are *s*_*E*_ = −0.019, *s*_*I*_ = −0.040, *D* = 0.0025, *g*_*EE*_ = 6, *g*_*IE*_ = *g*_*EI*_ = 2.8, *g*_*II*_ = 1, *g*_*gap*_ = 0.1, *τ*_*E*_ = 1, *τ*_*I*_ = 0.5, *κ*_*E*_ = 1, and *κ*_*I*_ = 1.
